# Persistent deNO*x* Ability of CaAl_2_O_4_:(Eu, Nd)/TiO_2-*x*_N_*y*_ Luminescent Photocatalyst

**DOI:** 10.1007/s11671-010-9750-7

**Published:** 2010-08-20

**Authors:** Huihui Li, Shu Yin, Tsugio Sato

**Affiliations:** 1 Institute of Multidisciplinary Research for Advanced Materials, Tohuko University, 2-1-1 Katahira, Sendai, Aoba-ku Japan

**Keywords:** Luminescent photocatalyst, deNO*x*, Composite

## Abstract

**Abstract:**

CaAl_2_O_4_:(Eu, Nd)/TiO_2-*x*_N_*y*_ composite luminescent photocatalyst was successfully synthesized by a simple planetary ball milling process. Improvement of photocatalytic deNO*x* ability of TiO_2-*x*_N_*y*_, together with the persistent photocatalytic activity for the decomposition of NO after turning off the light were realized, by coupling TiO_2-*x*_N_*y*_ with long afterglow phosphor, CaAl_2_O_4_:(Eu, Nd). The novel persistent photocatalytic behavior was related to the overlap between the absorption wavelength of TiO_2-*x*_N_*y*_ and the emission wavelength of the CaAl_2_O_4_:(Eu, Nd). It was found that CaAl_2_O_4_:(Eu, Nd)/TiO_2-*x*_N_*y*_ composites provided the luminescence to persist photocatalytic reaction for more than 3 h after turning off the light.

**Graphical Abstract:**

CaAl_2_O_4_:(Eu, Nd)/TiO_2-*x*_N_*y*_ composite luminescent photocatalyst with persistent deNO*x* activity after turning off the light was successfully synthesized by a simple planetary ball milling process. The novel persistent photocatalytic behavior was related to the overlap between the absorption wavelength of TiO_2-*x*_N_*y*_ and the emission wavelength of the CaAl_2_O_4_:(Eu, Nd).

## Introduction

Hot photocatalytic research attention has been focused on titania (TiO_2_), because of its chemical stability [1], excellent photocatalytic activity [2] and low cost. However, since titania has large band gap energy of about 3.2 eV corresponding to the wavelength of 387.5 nm, it is active under irradiation of only UV light less than 400 nm of wavelength. Since the content of UV light in sun light is less than 5% [3], the development of high performance visible light responsive photocatalyst which can use main part of sunlight or indoor light is highly desired [4-7]. Various modifications have been devoted to TiO_2_ in extending the absorption edge into visible light and enhancing the photocatalytic activity [8-13], and one of them is doping TiO_2_ with nitrogen because the band gap of titania could be narrowed by doping with nitrogen ion since the valence band of N2p band locates above O2p band [14].

The aluminate long afterglow phosphor (CaAl_2_O_4_:(Eu, Nd)) has characteristics of high luminescent brightness around 440 nm of wavelength, long afterglow time, good chemical stability and low toxicity [15,16]. Therefore, the coupling of TiO_2_ with CaAl_2_O_4_:(Eu, Nd) was expected to prolong the photocatalytic activity even after turning off the light by using the persistent emitting luminescence of the long afterglow phosphor as a light source of TiO_2_ photocatalyst. However, TiO_2_ possessing a large bandgap energy ca. 3.2 eV can not be effectively excited by the visible light luminescence of 440 nm from CaAl_2_O_4_:(Eu, Nd). Recently, the combinations of TiO_2_ photocatalyst with other long afterglow materials such as BaAl_2_O_4_:(Eu, Dy) [17] and Sr_4_Al_14_O_25_:(Nd, Eu) [18] were also reported. However, the emission wavelengths of these phosphors around 495 nm [19] and 488 nm, respectively, are also too long to excite TiO_2_ photocatalyst. Actually, it was reported that BaAl_2_O_4_:(Eu, Dy)/TiO_2_ and Sr_4_Al_14_O_25_:(Nd, Eu)/TiO_2_ coupled compounds showed photocatalytic performance for the oxidation of gaseous benzene and RhB solution, respectively, under UV light irradiation, but no noticeable degradation was observed after turning off the light [17].

In the present research, we firstly provided a direct evidence for such persistent photocatalytic deNO*x* system, by the coupling of long afterglow phosphor CaAl_2_O_4_:(Eu, Nd) with brookite type nitrogen-doped titania (TiO_2-*x*_N_*y*_), which was produced by a hydrothermal reaction [20,21]. Brookite phase nitrogen-doped titania possessed band gap of ca. 2.34 eV and showed excellent photocatalytic deNO*x* ability even under visible light irradiation of wavelength >510 nm [20]. In comparison with anatase and rutile phase nitrogen-doped titania, brookite phase nitrogen-doped titania photocatalyst has seldom been reported, however, it is expected to be a potential novel photocatalyst.

## Experimental Section

CaAl_2_O_4_:(Eu, Nd) powders with the particle size of 13.9 μm (D_50_) were purchased from Nemoto Co. Ltd. Other chemicals were purchased from Kanto Chem. Co. Inc. Japan and were used as received without further purification. TiO_2-*x*_N_*y*_ nanoparticles with brookite phase were synthesized by hydrothermal reaction using TiCl_3_ as titanium source and HMT (hexamethylenetetramine) as nitrogen source at pH 7 and 190°C for 2 h [20]. Brookite phase TiO_2-*x*_N_*y*_ nanoparticles were mixed with desired amounts of CaAl_2_O_4_:(Eu, Nd) powders followed by planetary ball milling at 200 rpm for 20 min. The mass ratio of CaAl_2_O_4_:(Eu, Nd):TiO_2-*x*_N_*y*_ or P25 TiO_2_ was kept at 3/2. For comparison, undoped titania (Degussa P25) was also coupled with CaAl_2_O_4_:(Eu, Nd) by the completely same manner. The UV–vis diffuse reflectance spectra were obtained using a UV–vis spectrophotometer (Shimadazu, UV-2450). The time dependence of photoluminescence spectra and intensity were measured by a spectrofluorophotometer (Shimadzu RF-5300P).

The photocatalytic activity for nitrogen monoxide destruction was determined by measuring the concentration of NO gas at the outlet of the reactor (373 cm^3^ of internal volume) during the photo-irradiation of a constantly flowing 1 ppm NO/50 vol% air mixed (balance N_2_) gas (200 cm^3^min^-1^). 0.16 g of CaAl_2_O_4_:(Eu, Nd)/TiO_2-*x*_N_*y*_, TiO_2-*x*_N_*y*_ or CaAl_2_O_4_:(Eu, Nd)/P25 photocatalyst material was placed in the same area of a hollow of 40 × 30 × 0.5 mm on a glass holder plate and set in the bottom center of the reactor. A 450 W high-pressure mercury lamp was used as the light source, where the inner cell had water flowing through a Pyrex jacket between the mercury lamp and the reactor. The light of λ < 290 nm wavelength was cut off by Pyrex glass [20-22]. Before light irradiation, the NO gas was continuously flowed through the reactor for 10 min to achieve adsorption balance. Then, the light was irradiated for 30 min to realize the steady status of the photocatalytic NO degradation and let long afterglow phosphor CaAl_2_O_4_:(Eu, Nd) absorb enough exciting energy. After that, the light was switched off, while the NO gas was flowed further for 3 h.

## Results and Discussion

**Figure 1:**
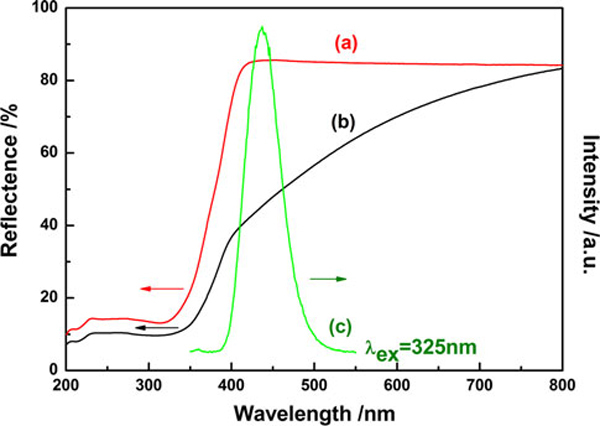
**Overlap of diffuse reflectance spectra of a undoped TiO_2_(P25) and b TiO_2-*x*_N_*y*_ and c emission spectrum of CaAl_2_O_4_:(Eu, Nd)**.

Figure 1 shows the diffuse reflectance spectra of undoped and nitrogen-doped titania and the emission spectrum of CaAl_2_O_4_:(Eu, Nd). CaAl_2_O_4_:(Eu, Nd) emitted blue luminescent light with a peak of 440 nm in wavelength by UV light irradiation (325 nm). Although undoped titania absorbed only UV light of the wavelength less than 400 nm, nitrogen–doped titania showed absorption of visible light up to 700 nm showing a nice overlap between the diffuse reflectance spectrum of TiO_2-*x*_N_*y*_ and the emission spectrum of CaAl_2_O_4_:(Eu, Nd). Therefore, it implied the potential possibility of CaAl_2_O_4_:(Eu, Nd)/TiO_2-*x*_N_*y*_ composite as the luminescent assisted photocatalyst which use the long after glow from the phosphor as the light source of the photocatalyst. Our previous research proved that nitrogen doped titania could be induced the photocatalytic activity by such weak LED light as 2.0 mW/cm^2^ with long wavelength of 627 nm [23,24]. This result also strongly implied the potential application of the composite as luminescent assisted photocatalyst material.

**Figure 2:**
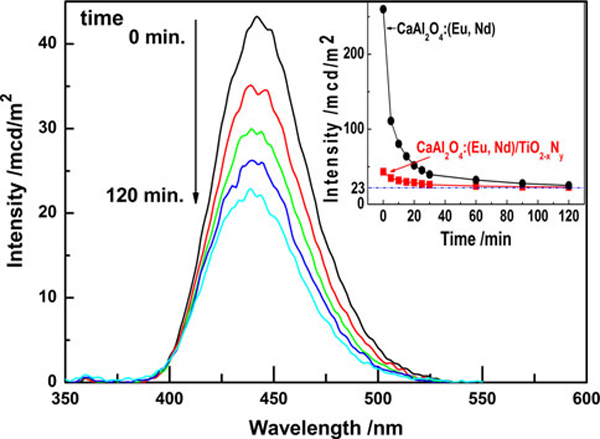
**The emission decay profile of CaAl_2_O_4_:(Eu, Nd)/TiO_2-*x*_N_*y*_ composite after irradiation by the mercury lamp used for photocatalytic reactions**. The inset shows the decline of the intensity of the emission.

Figure 2 shows the emission decay profile of CaAl_2_O_4_:(Eu, Nd)/TiO_2-*x*_N_*y*_ composite. The composite showed an emission spectrum peaked at 440 nm, which was almost identical to that of CaAl_2_O_4_:(Eu, Nd), attributed to the typical 4f^6^5d^1^-4f^7^ transition of Eu^2+ ^[16]. This indicated that the even if 40% brookite TiO_2-*x*_N_*y*_ was coated on the surface of CaAl_2_O_4_:(Eu, Nd) particles, comparatively strong luminescence property of the composite was kept. Although the emission intensity decayed with time, the emission intensity about 23 mcd/mm^2^ was retained even after 2 h.

**Figure 3:**
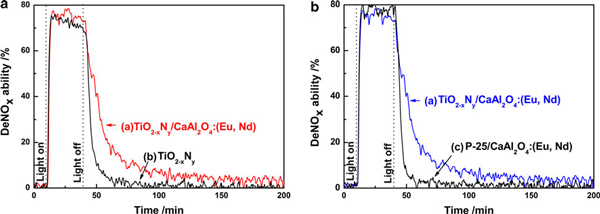
**The photocatalytic deNO*x* activity of the prepared samples during UV light irradiation for 30 min followed by turning off light, while NO gas was continuously flowed in the dark for 3 h**. **a** CaAl_2_O_4_:(Eu, Nd)/TiO_2-*x*_N_*y*_ composite; **b** brookite phase TiO_2-*x*_N_*y*_; **c** CaAl_2_O_4_:(Eu, Nd)/undoped TiO_2_ (P25) composite.

Figure 3 shows the photocatalytic NO destruction behaviors of CaAl_2_O_4_:(Eu, Nd)/TiO_2-*x*_N_*y*_, TiO_2-*x*_N_*y*_ and CaAl_2_O_4_:(Eu, Nd)/undoped TiO_2_ (P25) under UV light irradiation and after turning off the light. It was obvious that all the samples possessed excellent photocatalytic deNO*x* activity under UV light irradiation. Although the effect was very limited, it could be actually confirmed from the data of Figure 3a, b that under irradiation of high pressure mercury lamp (The data between *light on* and *light off*), CaAl_2_O_4_:(Eu, Nd)/TiO_2-*x*_N_*y*_ luminescent photocatalyst exhibit better photocatalytic activity than that of TiO_2-*x*_N_*y*._

The characterization system used in the present research was similar to that of the Japanese Industrial Standard which was established at the beginning of 2004 [25]. In this JIS standard, it is recommended that the photocatalytic activity of photocatalyst should be characterized by measuring the decrease in the concentration of NO at the outlet of a continuous reactor. One ppm of NO gas with a flow rate of 3.0 dm^3^/min is introduced to a reactor then irradiated by a lamp with light wavelength of 300–400 nm. The mechanism of photocatalytic deNO*x* had been researched carefully by M.Anpo [26]. During the deNO*x* photocatalytic reaction, the nitrogen monoxide reacts with these reactive oxygen radicals, molecular oxygen, and very small amount of water in air to produce HNO_2_ or HNO_3_. It was confirmed that about 20% of nitrogen monoxide was decomposed to nitrogen and oxygen directly [26] Because a continuous reaction system was utilized in the deNO*x* characterization [20,21], after turning off the light, it took about 10 min (total 50 min from the start of the characterization) to achieve diffusion balance and return to the initial NO concentration.

The degree of NO destruction by TiO_2-*x*_N_*y*_ and CaAl_2_O_4_:(Eu, Nd)/undoped TiO_2_ (P25) immediately decreased after turning off the light, however, as-expected, CaAl_2_O_4_:(Eu, Nd)/TiO_2-*x*_N_*y*_ retained the NO destruction ability for about 3 h. Since the decay profile of the NO destruction degree of CaAl_2_O_4_:(Eu, Nd)/TiO_2-*x*_N_*y*_ was similar to the emission decay profile shown in Figure 2, it might be concluded that the emission by CaAl_2_O_4_:(Eu, Nd) was used as a light source to excite TiO_2-*x*_N_*y*_ photocatalyst. It was also confirmed that CaAl_2_O_4_:(Eu, Nd)/TiO_2-*x*_N_*y*_ composite consisted of 40% brookite TiO_2-*x*_N_*y*_ (mass ratio of CaAl_2_O_4_:(Eu, Nd)/TiO_2-*x*_N_*y*_ = 3/2) possessed the best performance after turning off the light.

Present results indicate that the combination of CaAl_2_O_4_:(Eu, Nd) and TiO_2-*x*_N_*y*_ is a key point to realize the persistent catalytic activity even after turning off the light. In addition, it is well known that the combination of the two different band structure compounds may cause the charge transfer on the photocatalyst surface to depress the recombination of photo-induced electrons and holes, which is helpful for the improvement of photocatalytic activity [27,28]. This novel system provides a possibility of atmosphere purification not only in day time, but also in night time. A promising strategy involves coupling of visible light induced photocatalyst with long afterglow phosphor might be established. It is a new concept for the photocatalyst synthesis and applications.

## Conclusion

A novel CaAl_2_O_4_:(Eu, Nd)/TiO_2-*x*_N_*y*_ composite luminescent photocatalyst was successfully synthesized. Not only the UV-light induced photocatalytic activity, but also the persistent catalytic ability after turning off the light was realized successfully. The CaAl_2_O_4_:(Eu, Nd)/TiO_2-*x*_N_*y*_ composite photocatalyst provided enough luminescence intensity for the photocatalytic reaction for more than 3 h after turning off the light source.

## Supplementary Material

Additional file 1 Click here for file
