# Supplementary material for: Persistent deNOx Ability of CaAl2O4:(Eu, Nd)/TiO2-xNy Luminescent Photocatalyst
Source: Nanoscale Res Lett. 2010 Aug 20;6(1):5. doi: 10.1007/s11671-010-9750-7 (PMC3102338; doi:10.1007/s11671-010-9750-7)
Supplement: Additional file 1 [file 1556-276X-6-5-S1.doc]

**Electronic Supplementary Information (ESI)**

**Persistent DeNOx ability of CaAl_2_O_4_:(Eu, Nd)/ Luminescent Photocatalyst**

Huihui Li, Shu Yin* and Tsugio Sato

Institute of Multidisciplinary Research for Advanced Materials, Tohoku University,

Sendai, 980-8577, Japan

*E-mail: shuyin@tagen.tohoku.ac.jp

The morphology of the CaAl_2_O_4_:(Eu, Nd)/TiO_2-x_N_y_ luminescent photocatalyst were observed by a field emission scanning electron microscope (FESEM, Hitachi, S4800) and a transmission electron micrograph (TEM, JEOL JEM-2010). Fig.SI-1 shows the images of the long afterglow phosphor and its composite with nitrogen doped titania nanoparticles. It is obvious that CaAl_2_O_4_:(Eu, Nd) phosphor sample possessed large particle size and smooth surface(Fig.(a)), while the CaAl_2_O_4_:(Eu, Nd)/TiO_2-x_N_y_ composite consisted of TiO_2-x_N_y_ nano-particles on the surface of the samples, indicating the formation of composition structure of the luminescent photocatalyst. The TEM image also agreed with the fact that observed from the FESEM images, i.e., large particles of CaAl_2_O_4_:(Eu, Nd) were surrounded by brookite TiO_2-x_N_y_ nanoparticles with 15-20 nm in size. In TEM observation, comparative small particle size of the long afterglow phosphor (1.5 μm) was related to the sample gather conditions using Cu grid.


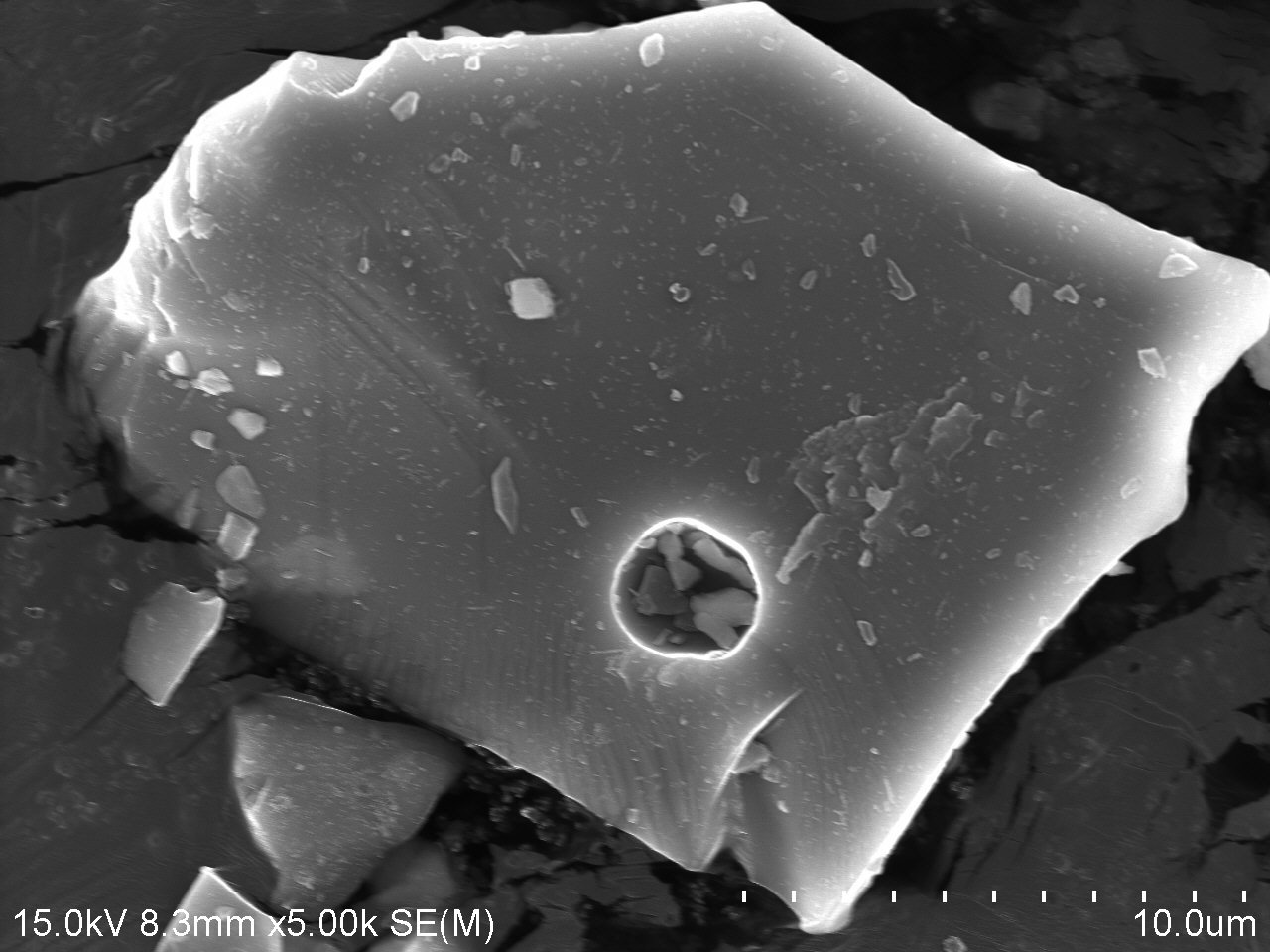


**(a)**


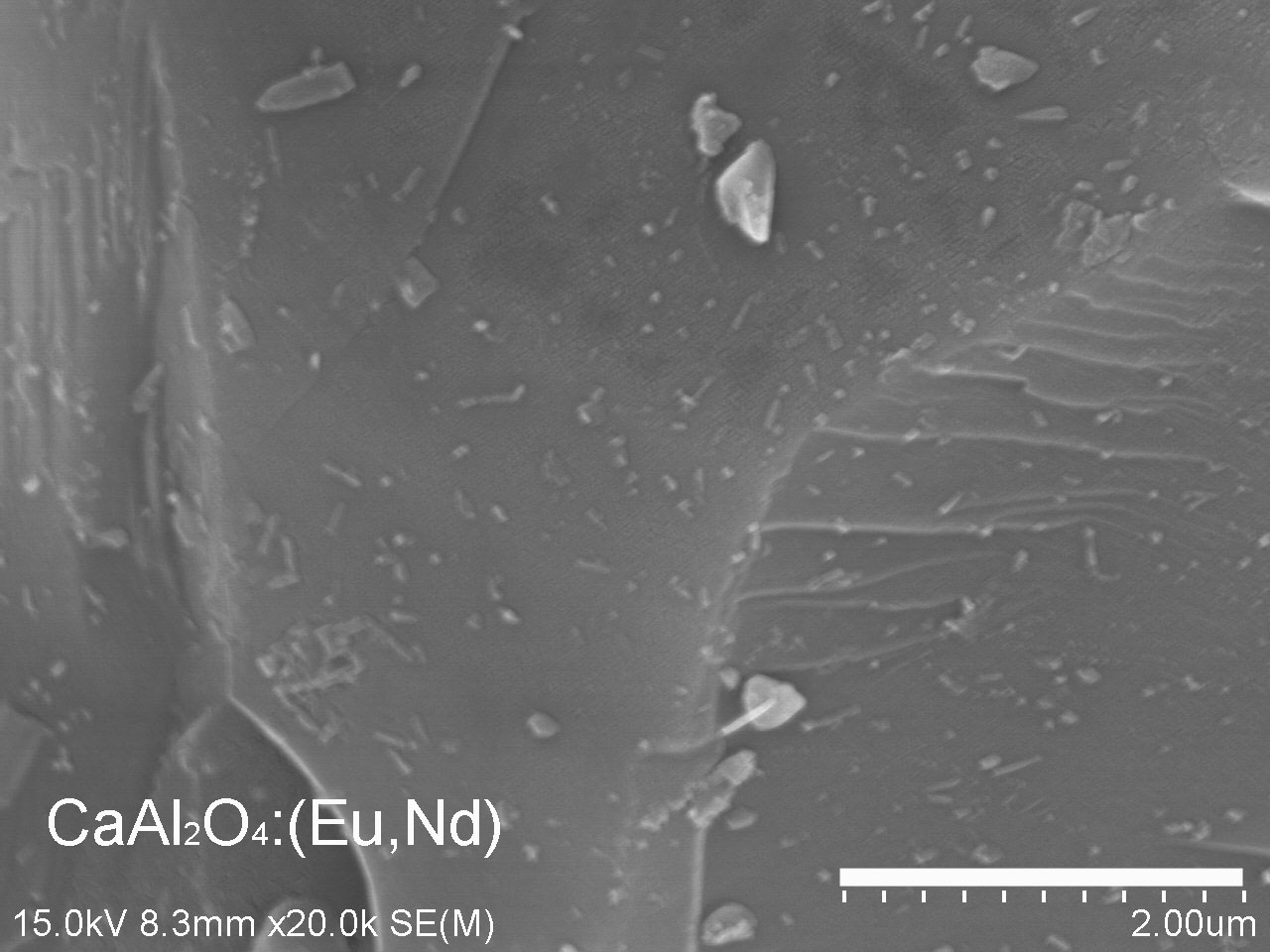


**(a)**


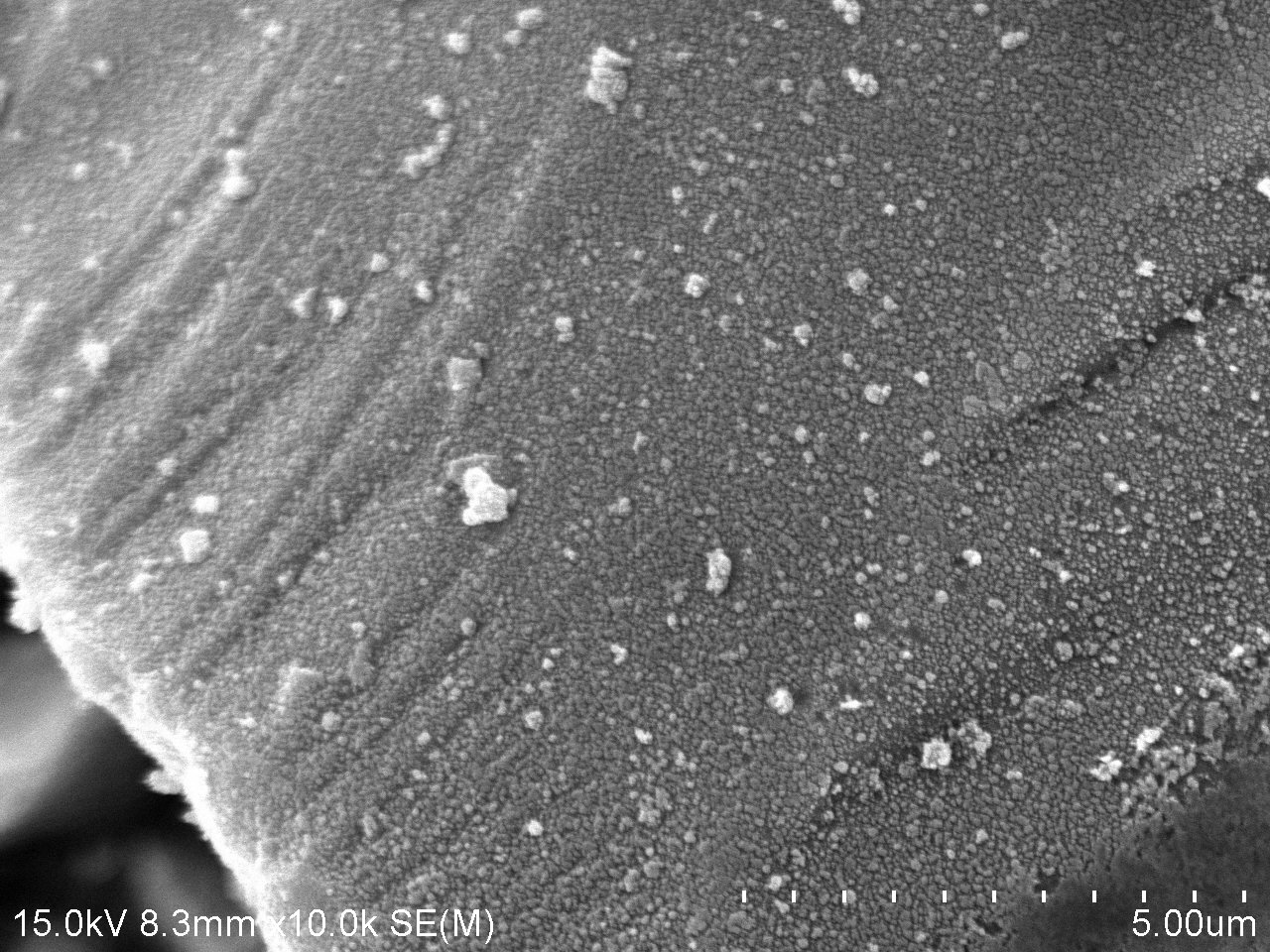


**(b)**


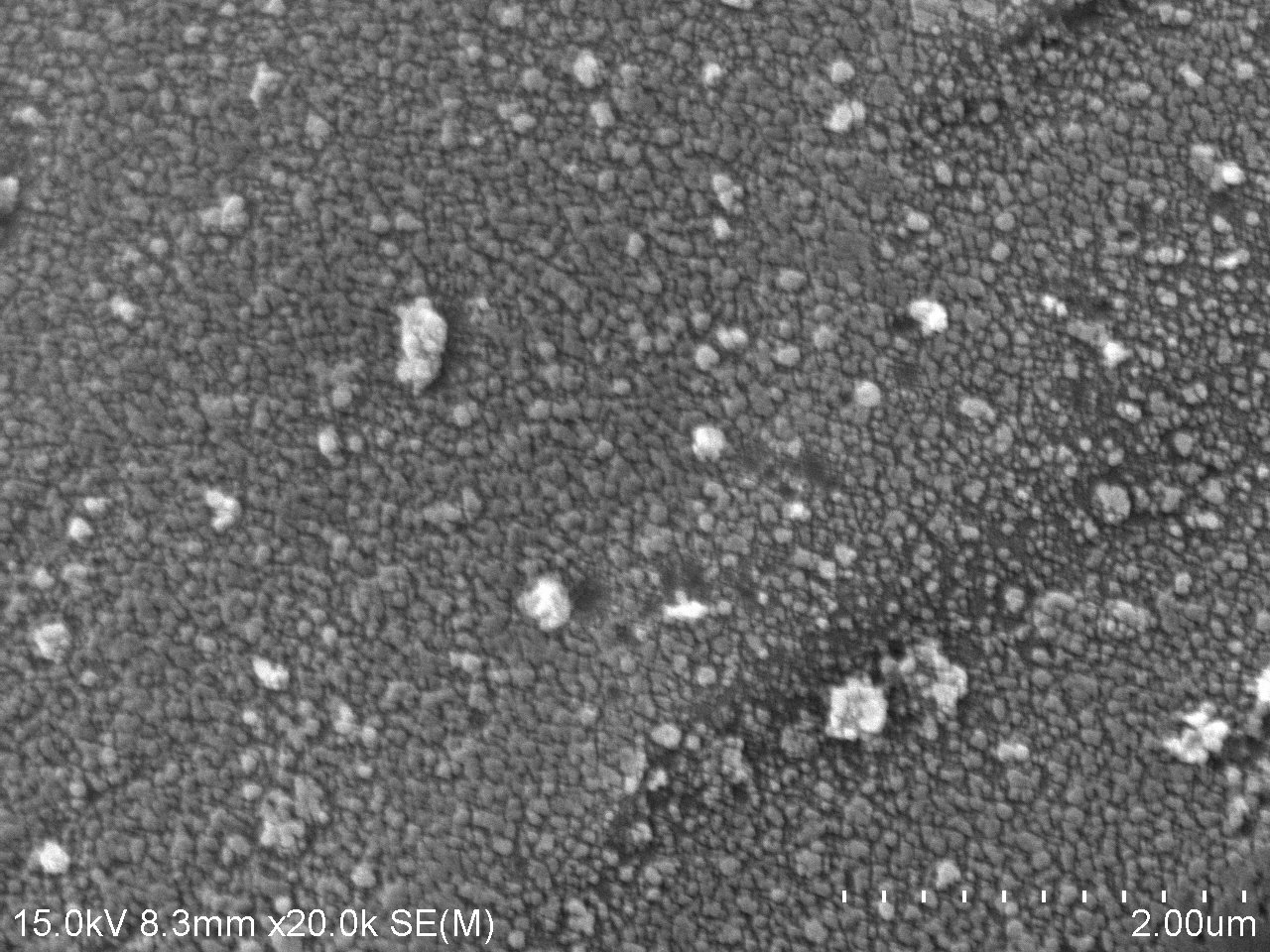


**(b)**


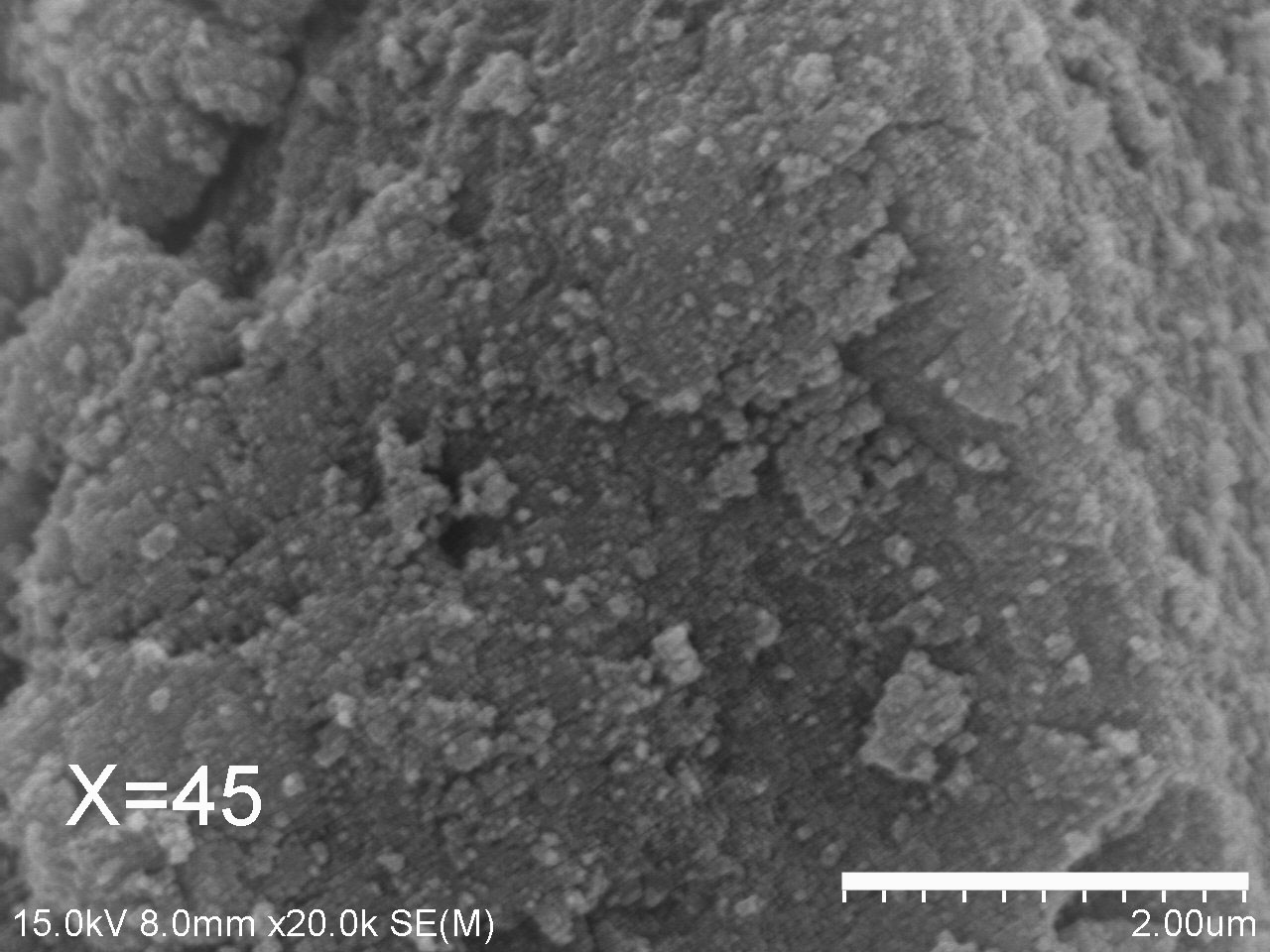


**(b)**


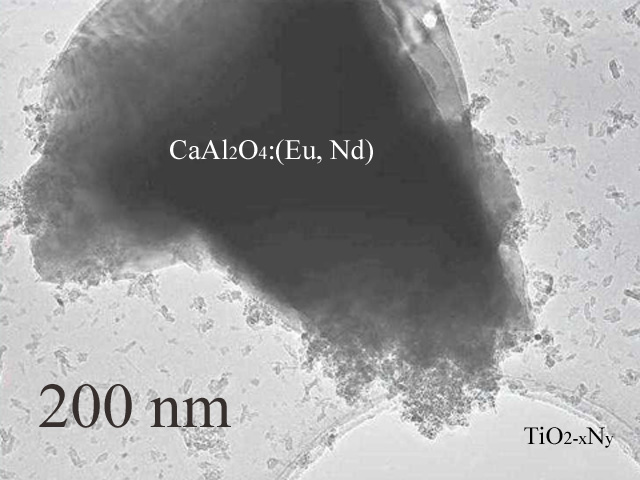


**(b)**

Figure SI-1. FESEM and TEM photographs the (a) CaAl_2_O_4_:(Eu, Nd) and (b) CaAl_2_O_4_:(Eu, Nd)/TiO_2-x_N_y_ composite particles.
